# 

**Published:** 1888-12

**Authors:** 


Volume LVII.] DECEMBER, 1888. [Number 6.

### The CHICAGO MEDICAL JOURNAL AND EXAMINER

EDITOR:
S. J. JONES, M.D., LL.D.

PUBLISHED MONTHLY UNDER THE AUSPICES OF
THE CHICAGO MEDICAL PRESS ASSOCIATION.

RAND, McNALLY & CO., PRINTERS.

Entered at the Post Office at Chicago, for transmission through the mails as second-class matter.

